# Patient Comfort With Smartphone-Based and Intraoral Camera Imaging in Teledentistry: Observational Cross-Sectional Study

**DOI:** 10.2196/95563

**Published:** 2026-08-05

**Authors:** Martha B Lucas, Boyen Huang, Ryan HL Ip, Angela M Hastings, Emily C Schultz, Yao-Yi Chiang, Jeffrey P Louie

**Affiliations:** 1 Department of Primary Dental Care School of Dentistry University of Minnesota Minneapolis, MN United States; 2 Department of Mathematical Sciences Auckland University of Technology Auckland, Auckland New Zealand; 3 Department of Dental Education College of Allied Health and Nursing Minnesota State University, Mankato Mankato, MN United States; 4 Department of Computer Science & Engineering College of Science and Engineering University of Minnesota Minneapolis, MN United States; 5 Department of Pediatrics Medical School University of Minnesota Minneapolis, MN United States

**Keywords:** mobile health, mHealth, patient comfort, tooth structure loss, crown fracture, caries, tooth wear

## Abstract

**Background:**

Smartphone-based imaging has shown promise in teledentistry; yet, patient comfort during its use compared with intraoral cameras remains underexplored.

**Objective:**

This study aimed to compare self-perceived comfort during oral evaluations using smartphones and intraoral cameras in a teledentistry setting.

**Methods:**

A convenience sample of 192 participants aged 6 years and older completed a survey and underwent 2 procedures: trained researchers captured 3 photographs of the anterior teeth with a Samsung smartphone, and the same teeth were examined with a MouthWatch intraoral camera. Comfort was rated by participants before and after each procedure using a pictorial 7-point Likert scale, and data were analyzed with nonparametric tests.

**Results:**

Comfort decreased in 15% (29/192) of the participants following intraoral camera examination (*P*=.001), while no significant change was observed after smartphone imaging (*P*=.52). The difference in comfort change between smartphone imaging and intraoral camera examination was marginal (*P*=.05). Among the small subgroup of youths (aged 6-19 years; n=13, 6.8%), 38% (5/13) reported a reduction in comfort after smartphone imaging, compared with 10% (18/179) among adults (*P*=.02). In addition, significantly more participants without tooth wear reported a reduction in comfort after smartphone imaging (5/12, 42%), compared with 10% (18/180) among those with tooth wear (*P*=.02).

**Conclusions:**

Overall, the findings indicate generally comparable comfort levels between the 2 approaches, with a marginal difference favoring smartphone imaging. While the potential for broader use of smartphone-based imaging in a teledentistry setting still requires further research, targeted education and aligned institutional and insurance policies may enable its scalable, sustainable integration that enhances patient experience.

## Introduction

Teledentistry uses digital technologies to deliver synchronous and asynchronous oral health care and dental assessments to patients who are not physically present with dental professionals. Initially developed to support dental consultations for US troops, teledentistry helped improve access to care and reduce travel burdens within military settings [1]. In addition to expanding access, teledentistry has been promoted as a strategy to reduce oral health inequalities and lessen the financial impact associated with traditional dental care [2]. Teledentistry models vary, with some enabling real-time interactions between providers and patients through video consultations and others relying on photographs captured for clinical evaluation [3]. In many cases, intraoral cameras are used to obtain images of the oral cavity for remote assessment [4-6]. More recently, teledentistry has expanded into mobile health apps, in which patients or caregivers use smartphone cameras to take photographs that are later reviewed by dental professionals. Studies have demonstrated the feasibility of remote assessment of dental caries [3], traumatic dental injuries [7], and dental restoration quality [8] using smartphone-based imaging. Similarly, the feasibility and reliability of teledentistry assessments for dental caries [9] and oral potentially malignant disorders [10] using images captured with intraoral cameras have been confirmed. Despite this potential, access to in-person dental care and diagnosis remains limited for many individuals, especially those in low-income, uninsured, and remote communities who face a disproportionately high burden of oral disease [11]. Because traditional diagnostic visits can be costly and time-intensive, alternative approaches are needed. Teledentistry provides a means of remote assessment and patient triage, improving access to care while reducing demand on conventional dental settings [12].

Although evidence supports the diagnostic accuracy of teledentistry, barriers to adoption persist. While many dental professionals express support for teledentistry [13,14], patient acceptance varies considerably. Reported barriers include the absence of physical contact during examinations [15], perceived psychological, financial, and performance risks [16], limited access to digital devices or limited digital literacy [15], concerns about privacy and confidentiality [17], and discomfort with capturing intraoral photographs [15]. Social influences also play a role, with greater acceptance observed when family and peers endorse teledentistry [16], and patient anxiety during procedures further affects compliance and outcomes [18]. Together, these factors underscore that patient comfort and acceptance are critical for the successful integration of teledentistry into routine care.

While both smartphones and intraoral cameras are increasingly used for the detection of dental caries and oral cancer [4-6], most comparative research has centered on diagnostic accuracy [5,6]. Far less attention has been given to patient-centered outcomes, particularly comfort and acceptance. To date, only one study has examined children’s acceptance of both modalities during caries screening [4], highlighting a notable gap in understanding how adolescents and adults experience these tools in terms of emotional and physical comfort. In this context, comfort refers to a state of ease marked by the absence of distress and the presence of safety, positivity, and situational acceptance [19]. Research in other clinical fields has documented patient comfort with mobile imaging technologies, such as smartphone-based photography in neurology [20] and portable otoscope telemedicine devices in otology [21]. Within teledentistry, however, only one recent study has evaluated patient comfort with smartphone-based imaging from the perspective of dental clinicians, and it did not directly capture patients’ own interpretations of comfort [17].

To address this gap, this study aimed to assess prospective patients’ comfort levels during examinations conducted with an intraoral camera versus those involving smartphone-captured photographs. The goal was to generate evidence that could guide strategies for expanding the use of teledentistry among the general population and, more specifically, to support broader access to dental care in remote and underserved communities. Accordingly, this study tested whether patient comfort differed between the 2 imaging modalities.

## Methods

### Ethical Considerations

This study was approved by the institutional review board of the University of Minnesota (STUDY00022279). Informed consent was obtained from all adult participants, and parental consent was obtained for those younger than 18 years. All study personnel followed institutional review board–approved procedures for data access, confidentiality, and privacy. All data were anonymized and deidentified prior to analysis. Participants received a spin toothbrush as compensation after providing consent and completing the study procedures.

### Study Design and Setting

This study adhered to the STROBE (Strengthening the Reporting of Observational Studies in Epidemiology) guideline for cross‑sectional studies [22]. The observational cross-sectional study was conducted in Minnesota, United States, in 2024, as part of a multisite machine learning project designed to advance teledentistry technology and optimize user experience for image-based assessments of tooth structure loss (TSL), encompassing crown fractures, caries, and tooth wear [23]. Researchers used the Minnesota State Fair as a community-based setting to recruit prospective dental patients who may or may not have had immediate dental care needs. Recruitment and data collection took place inside the University of Minnesota’s Driven to Discover building, a designated research facility located on the fairgrounds that provided space for fairgoers and researchers to come together for study participation and community engagement. The Minnesota State Fair is an annual ticketed event featuring agricultural exhibitions, art, food, entertainment, and carnival rides. Every year, it attracts approximately 2 million fairgoers from across the country over 12 days from late August to early September [24], offering a diverse pool of potential participants.

### Participants

To support a separate machine learning analysis using the same dataset, approximately 150 dental photos were needed for each TSL condition [25], contributing to a cumulative target of 600 participants. Recruitment was planned across multiple sites, with at least 200 participants enrolled at each, including the Minnesota State Fair and 2 international collaborating locations. Eligibility criteria required participants to be at least 6 years old, exhibit one or more signs of TSL, and be able to communicate in English.

Recruitment followed a standardized procedure. Researchers were stationed at the entrance of the Driven to Discover building during designated data collection hours (9 AM-8 PM on 3 separate days). Passersby were visually screened for potential eligibility based on age and the presence of visible signs of TSL. Researchers used a brief, scripted verbal explanation to invite eligible individuals to participate. Additional participants self-referred after viewing a signboard indicating that participation included receiving a spin toothbrush. Participants with identifiable features in or around the mouth (eg, piercings, birthmarks, tattoos, or jewelry) were excluded. Given the open and recreational nature of the fair environment, convenience sampling was used.

### Devices and Materials

Dental photos were taken using the camera function of a Samsung Galaxy S20 FE 5G smartphone, without any image editing, and then securely uploaded to the Box cloud storage system. These smartphones were purchased new for our previous study [7], restricted to password-protected institutional Wi-Fi (eduroam), prohibited from installing additional apps, and stored and managed by a key researcher. Individual user accounts and passwords were required for Box. MouthWatch intraoral cameras were used to assess the participants’ anterior teeth and to complete a dental chart, which also operated independently of linked software. A survey tool, designed to collect demographic information and evaluate comfort levels, was administered using iPads (Apple Inc). Additionally, a dental chart was accessed through the Notability app on the iPad and later transferred to a spreadsheet for auditing and analysis.

### Procedure

All researchers were trained and calibrated before participant recruitment and data collection. Each participant completed a self-administered demographic questionnaire. Using a pictorial version of a 7-point Likert scale [26], participants rated their comfort levels before and after each imaging method. Examinations were conducted in a fixed sequence, beginning with the intraoral camera and followed by smartphone imaging with flash. Due to the recreational and high‑throughput nature of the Minnesota State Fair setting, procedural simplicity was required in the affiliated research facilities; therefore, the order of modalities was not randomized or counterbalanced.

**Figure 1:**
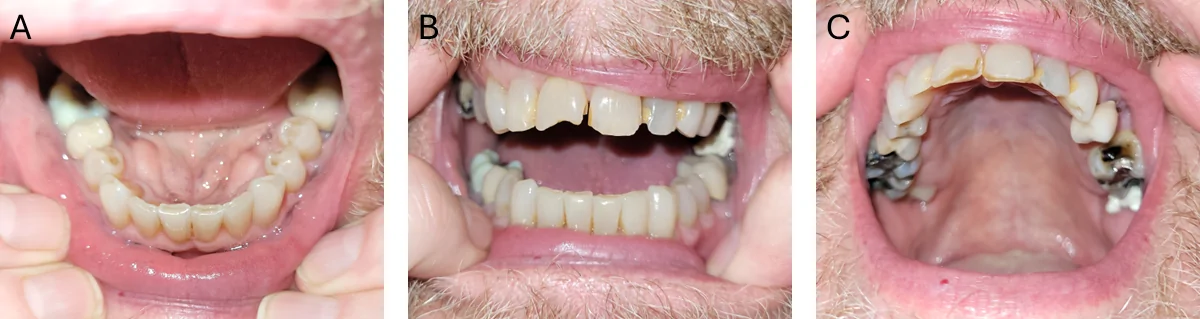
Example dental photographs captured during the study using a Samsung Galaxy S20 FE 5G smartphone in a cross-sectional teledentistry evaluation of tooth structure loss among fairgoers at the 2024 Minnesota State Fair (United States): (A) mandibular occlusal view, (B) frontal view, and (C) maxillary occlusal view. For infection-control purposes, participants retracted their lips with their own fingers during image capture. The images are shown with the participant’s consent.

Consistent with prior publications using smartphone imaging in dentistry, 3 standardized photographs were captured for each participant (Figure 1) [7]. Images were taken with the designated smartphone under natural light with the flash feature activated. Each participant was seated upright in a chair, and the researcher stood directly in front during image capture. All images were immediately uploaded via a secure wireless network to the Box cloud system. An experienced teledentist subsequently performed a remote assessment, charting tooth-by-tooth conditions, including uncomplicated and complicated crown fractures, caries, tooth wear, and teeth that were missing, restored, sound, or partially erupted.

### Data Analysis

Data entry and statistical analysis were performed using Excel (Microsoft Office LTSC Professional Plus 2024) and R (version 4.3.3; R Foundation for Statistical Computing), respectively. Counties of residence were categorized as either “metropolitan” or “nonmetropolitan” according to the 2023 Rural-Urban Continuum Codes [27].

Due to the subjective nature of the comfort levels, the “before and after differences,” defined as the comfort level after a photo is taken minus that before a photo is taken for the same participant, were used as the outcome variables to reduce the effects of within-participant variability. The changes in comfort levels after each device were assessed among all participants, as well as among different subgroups formed by potential confounding variables such as demographics and dental conditions. The changes in comfort levels across all participants were analyzed using the Wilcoxon signed-rank test. The changes in comfort levels between different subgroups were analyzed using the Wilcoxon rank-sum test (when there were 2 subgroups) and Kruskal-Wallis test (when there are 3 or more subgroups). The use of nonparametric methods was appropriate due to the nonnormal nature of the data collected. The level of significance was set at 5%.

## Results

226 fairgoers consented to participate. After excluding participants with missing data, a total of 192 (85%) participants were included in the final sample. These included 181 (94.3%) residing in Minnesota and 11 (5.7%) from other US states. The teledentist’s remote assessment confirmed TSL conditions (crown fractures, caries, or tooth wear) in 190 (99%) participants. Table 1 presents the frequency distribution of the final sample.

**Table 1:** Frequency distribution of demographic and dental characteristics and associated changes in comfort levels among 192 participants in an observational cross‑sectional teledentistry study conducted at the 2024 Minnesota State Fair (United States), including *P* values for comfort change after smartphone imaging, after intraoral camera examination, and between modalities.

				Frequency, n (%)			Comfort level change after smartphone imaging, *P* value			Comfort level change after intraoral camera examination, *P* value			Difference in comfort level change between smartphone imaging and intraoral camera examination, *P* value
**Demographics**													
	**Gender**					.69			.19			.32	
		Woman	98 (51)										
		Man	92 (48)										
		Nonbinary	2 (1.0)										
	**Race**					.30			.68			.93	
		Black, Indigenous, and People of Color	30 (15.6)										
		White	162 (84.4)										
	**Age (y)^a^**					.25			.20			.44	
		6-19	13 (6.8)										
		20-29	25 (13)										
		30-39	17 (8.9)										
		40-49	24 (12.5)										
		50-59	36 (18.7)										
		60-69	51 (26.6)										
		70-84	26 (13.5)										
	**Age group**					.02^b^			.32			.08	
		Adolescents	13 (6.8)										
		Adults	179 (93.2)										
	**Area of residence**					.76			.08			.10	
		Metropolitan	174 (90.6)										
		Nonmetropolitan	18 (9.4)										
**Dental conditions**													
	Caries					.18			.26			.80	
		Caries	32 (16.7)										
		No caries	160 (83.3)										
	**Crown fracture**					.86			.53			.34	
		Crown fracture	158 (82.3)										
		No crown fracture	34 (17.7)										
	**Tooth wear**					.02^b^			.80			.03^b^	
		Tooth wear	180 (93.8)										
		No tooth wear	12 (6.2)										
	**Restoration**					.85			.15			0.2	
		Restoration	164 (85.4)										
		No restoration	28 (14.6)										
		Overall^c^	192 (100)		.52			.001^b^			.05		

^a^Data analysis based on a Kruskal-Wallis test.

^b^*P*<.05.

^c^Data analysis based on a Wilcoxon signed-rank test.

**Figure 2:**
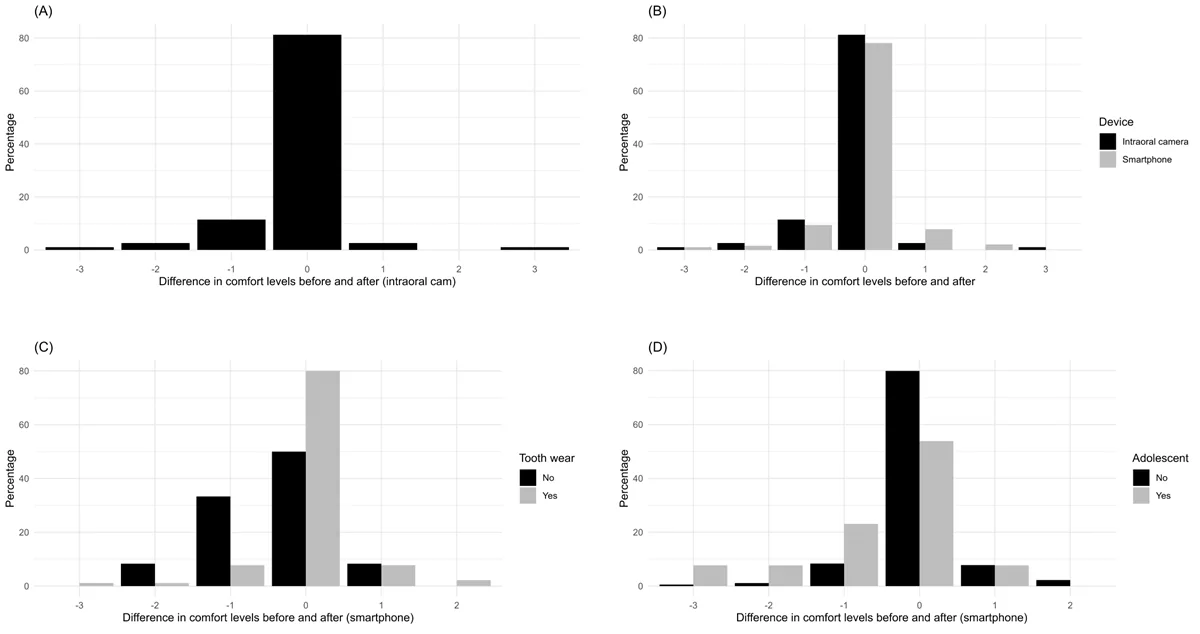
Changes in participant comfort levels before and after imaging procedures in an observational cross‑sectional study of teledentistry conducted at the 2024 Minnesota State Fair (United States): (A) comfort change after intraoral camera examination, (B) comparison of comfort change between smartphone imaging and intraoral camera examination, (C) comfort change after smartphone imaging by tooth wear status, and (D) comfort change after smartphone imaging by age group.

More participants reported a decrease in comfort levels than an increase following the intraoral camera examination (*P*=.001; Figure 2A). The result of the Wilcoxon signed-rank test reflects the asymmetry of the distribution. Of the 192 participants, 29 (15.1%) reported a reduction in comfort after the intraoral camera procedure, while only 7 (3.6%) indicated an increase in comfort. No significant change in comfort was observed after smartphone imaging (*P*=.52). When comparing the 2 modalities, the change in comfort levels before and after the procedure showed only a marginal difference between smartphone imaging and intraoral camera examination (*P*=.05; Figure 2B).

Participants without tooth wear experienced a greater decrease in comfort levels after smartphone imaging compared with those with tooth wear (*P*=.02; Figure 2C). Among the 12 participants without tooth wear, 5 (41.7%) reported a reduction in comfort levels, while only 10% (18/180) of the participants with tooth wear reported the same. It should be noted that among the 12 participants without tooth wear, 4 (33.3%) were younger than 20 years, 1 (8.3%) had caries, 8 (66.7%) had uncomplicated crown fractures, and 2 (16.7%) had complicated crown fractures. Note that these categories are not mutually exclusive and therefore the sum of the reported counts is greater than 12. Moreover, children and adolescents (aged 6-19 years) experienced a more pronounced reduction in comfort after smartphone use than adults (*P*=.02; Figure 2D). Among the 13 children and adolescents, 5 (38.5%) indicated a reduction in comfort, while only 10.1% (18/179) of participants aged 20 years or older reported the same.

No significant differences in comfort levels were observed by gender, metropolitan or nonmetropolitan residence, or other oral conditions (*P*≥.08).

## Discussion

### Interpretation of Findings

This study aimed to compare patient comfort during dental evaluations conducted with smartphone-based imaging and intraoral camera examination. Consistent with this objective, the findings indicated that comfort decreased following intraoral camera use, whereas smartphone imaging did not produce a significant change. When comparing the 2 modalities, smartphone imaging demonstrated comparable comfort, with a trend toward greater comfort relative to intraoral camera examination. These results directly addressed the study aim and contributed new evidence to the limited literature on patient-centered outcomes in teledentistry.

The consistent comfort associated with smartphone imaging may be due to factors such as familiarity, patient friendliness, simplicity, and portability, as suggested by Aly et al [4]. Given the widespread use of smartphones for communication, education, and recreation [28], individuals may feel more at ease, less intimidated, and more comfortable when photographed with such devices [4]. Similar preferences have been observed in other areas of dentistry where less invasive digital technologies, such as digital impression systems, have replaced traditional impression techniques [29]. In those contexts, patients have favored digital methods due to increased efficiency, reduced chair time, and reduced discomfort associated with prolonged mouth opening [30]. Similarly, in esthetic dentistry, digital 3D modeling has been preferred over conventional approaches for its shorter treatment duration, enhanced comfort, and reliability [31]. Although Aly et al [4] noted that smartphone imaging elicited lower fear and better behavior among pediatric patients, they also found that intraoral camera procedures were completed more quickly [4]. However, patients undergoing intraoral camera examinations experienced difficulty maintaining mouth opening [4]. Thus, perceived familiarity, ease of use, and reduced duration of mouth opening during smartphone imaging may matter more than its brevity. Future investigations are warranted to examine how these advantages, individually or in combination, affect patient comfort.

The present study identified an association between age and patient comfort levels, with children and adolescents exhibiting a greater reduction in comfort following smartphone imaging compared with adults. This pattern contrasts with a prior study reporting more favorable behavior in children examined using a smartphone compared with an intraoral camera [4]. In our sample, a smaller proportion of adults than children reported decreased comfort after smartphone imaging, and this age-related finding should be interpreted cautiously given the small number of youth participants (n=13) and its exploratory nature. This observation may reflect the well-documented inverse association between age and dental fear, which is more prevalent among younger populations [32]. Fear of dental visits and avoidance behaviors are especially common in children and adolescents, who may have difficulty understanding the purpose of dental examinations, particularly within a research context. The introduction of smartphone technology in a diagnostic dental setting may have further compounded their discomfort. Together, elevated dental anxiety and limited familiarity with smartphone-based imaging in a clinical environment may explain the greater decline in comfort among younger participants. To better support pediatric patients during smartphone-based teledentistry, clinical communication should incorporate friendly, interactive explanations of the procedures and include opportunities for children to view the images captured of their own teeth.

Subgroup analyses revealed that participants without tooth wear experienced a greater decline in comfort after smartphone imaging than those with tooth wear. No associations were observed between changes in comfort and other dental conditions, including caries, uncomplicated crown fractures, or complicated crown fractures. As documented in the literature, the prevalence of tooth wear increases with age [33], suggesting that participants without tooth wear were more likely to be younger. This was reflected in the present study’s distribution: 4 (33.3%) of the 12 participants without tooth wear were younger than 20 years, markedly higher than the overall proportion of the entire sample (13/192, 6.8%). Given the inverse relationship between age and comfort levels observed in this study, the greater discomfort reported by participants without tooth wear may be confounded by age. Furthermore, most participants without tooth wear (n=12) presented with other TSL signs: 1 with caries, 8 with uncomplicated crown fractures, and 2 with complicated crown fractures. Dental anxiety, often triggered by the discovery of oral disease and the anticipated need for intervention [34], is associated with avoidance of dental visits and poorer health outcomes [35]. Increased distress among patients with untreated dental conditions and a heightened perceived need for care may explain the discomfort reported by those without tooth wear during smartphone imaging [18]. Because the subgroup without tooth wear was small, these findings should be considered exploratory and validated in future research with larger and more balanced samples.

No differences in comfort levels were observed based on participants’ gender, race, or area of residence. Although prior research has not specifically examined comfort responses to teledentistry imaging modalities across these demographic groups, earlier studies have identified associations between demographic factors and dental anxiety. For example, female children [36], those of Asian descent [37], or those residing in rural areas have been shown to exhibit higher levels of dental anxiety [36]. Nevertheless, these findings should not be extrapolated to infer similar patterns in comfort with smartphone or intraoral camera imaging. Recent work has instead highlighted factors such as “clear communication, informed consent, and assurances regarding privacy and data security” as key contributors to patient comfort during smartphone-based dental imaging [17]. These considerations point to communication quality as a contextual factor that may shape patient experience across demographic groups.

### Limitations

Recruitment at the Minnesota State Fair introduced several limitations to this study. Participation in a large-scale outdoor event required the purchase of an entry ticket and the physical ability to navigate the fairgrounds, which may have excluded individuals with limited financial resources or physical disabilities. Moreover, individuals with more severe forms of TSL, such as deep caries with cellulitis or complicated crown fractures accompanied by bleeding or pain, were unlikely to attend a recreational venue, resulting in probable underrepresentation of these conditions. The study’s inclusion criteria focused exclusively on TSL, thereby excluding individuals with other oral health conditions such as periodontal disease, oral cancer, or xerostomia.

The recreational nature of the state fair also required procedural simplicity. Consequently, the order of imaging modalities was fixed rather than randomized, with all participants undergoing intraoral camera examination first, followed by smartphone imaging. This lack of randomization may have introduced an order-effect bias [38], whereby the experience of the first procedure could influence perceived comfort during the second. Although the direction of such an effect cannot be determined from the present data, the potential for order-related influence remains an important methodological limitation. Future studies should randomize or counterbalance the order of procedures to minimize this bias.

This study also used a single smartphone model and a single intraoral camera model, which limits the generalizability of the findings. Future research should incorporate multiple device types and diverse clinical settings to strengthen external validity.

Some statistically significant results were derived from relatively small subgroup sample sizes. Therefore, caution is required when interpreting these findings, and future studies should aim to recruit larger and more balanced subgroup samples.

Finally, the use of a convenience sample limited the generalizability of the findings. Random sampling was not feasible in the state fair setting, and similar constraints would likely arise in clinical recruitment. To enhance representativeness in future research, sampling across multiple community or school settings may provide a more diverse and generalizable participant pool.

### Conclusions

Within the study limitations, these findings suggest that smartphone-based imaging may serve as a patient-friendly alternative to intraoral cameras in both clinical and teledentistry settings. Beyond the immediate comparison of comfort levels, the results indicate that smartphone imaging may warrant further evaluation as a scalable, accessible component of remote dental assessment, particularly in underserved or resource-limited communities. Integrating smartphone-based imaging into routine practice may reduce barriers related to equipment cost, portability, and patient acceptance. To facilitate broader adoption, targeted education for patients and dental professionals, along with supportive institutional and insurance policies, will be essential. Future research should explore implementation strategies, longitudinal patient experiences, and the role of smartphone imaging in comprehensive teledentistry workflows.

## Data Availability

The deidentified datasets generated or analyzed during this study are available from the corresponding author on reasonable request.

## Abbreviations

STROBE: Strengthening the Reporting of Observational Studies in Epidemiology
TSL: tooth structure loss
